# *Cinnamomum zeylanicum* extract has antidepressant-like effects by increasing brain-derived neurotrophic factor (BDNF) and its receptor in prefrontal cortex of rats

**Published:** 2021

**Authors:** Mona Aryanezhad, Mohammad Abdi, Sabrieh Amini, Kambiz Hassanzadeh, Elham Valadbeigi, Kaveh Rahimi, Esmael Izadpanah, Mohammad Raman Moloudi

**Affiliations:** 1 *Department of Biology, Sanandaj Branch, Islamic Azad University, Sanandaj, Iran*; 2 *Cancer and Immunology Research Center, Research Institute for Health Development, Kurdistan University of Medical Sciences, Sanandaj, Iran*; 3 *Cellular and Molecular Research Center, Research Institute for Health Development, Kurdistan University of Medical Sciences, Sanandaj, Iran*; 4 *Neurosciences Research Center, Research Institute for Health Development, Kurdistan University of Medical Sciences, Sanandaj, Iran*

**Keywords:** Depression, Mood disorders, Receptor tyrosine kinases, Gene expression, Hydroalcoholic extract of Cinnamomum

## Abstract

**Objective:**

Depression is one of the most common mood disorders. Considering the evidence on the effect of *Cinnamomum* on mood disorders, this study investigatedthe effect of hydroalcoholic extract of *Cinnamomum* (HEC) in an animal model of depression.

**Materials and Methods:**

Thirty-two male rats were selected and divided into four groups (n=8) including: control, depressed, and depressed treated with200 and 400 mg/kg HEC. Depression induction protocol was conducted in all groups except for the control group. Sucrose preference test (SPT) and forced swimming test (FST) were done to analyze the depression score. After four weeks, the animals brain cortex was removed and BDNF protein and tyrosine receptor kinase B (*TrkB*) gene expression levels were determined by ELISA and Real Time PCR, respectively.

**Results:**

The results of this study showed that 400 mg/kg of HEC increased the tendency to drink the sucrose solution. Furthermore, immobility time significantly increased in the depressed group compared to the control group while it was attenuated by administration of 400 mg/kg extract on the 28th day versus the depressed group. Also the extract at both doses increased swimming time compared to the depressed group. In addition, an increase in the BDNF protein and *TrkB *gene expression levels was observed in the prefrontal cortex of the treatment groups.

**Conclusion:**

We found that HEC ameliorated depression symptoms in rats and these effects were probably due to an increase in BDNF proteins and its receptor, *TrkB*, gene expressions in the prefrontal cortex.

## Introduction

Depression is one of the most common mood disorders which is characterized by a depressed mood or feelings of sadness, low self-confidence, and lack of interest in daily activities, and pleasure (Mansson, 2019 ▶). It has been predicted that depression may be the second leading cause of death after heart disease by 2020 (Carney and Freedland, 2017 ▶). The prevalence of this disease in America is 16.9%, 3%in Japan (Andrade et al., 2003 ▶), 1.1% in Taiwan, 15.4% in the Netherlands (Steptoe et al., 2007 ▶), and 5.69–13% in Iran (Montazeri et al., 2013 ▶). Many hypotheses about the pathophysiology of depression and mood disorders have been proposed (Moinuddin et al., 2012 ▶; Patro et al., 2016 ▶). In monoamine theory, depression is a monoamine dysfunction that causes mood disorders, which is also based on the neurotrophic hypothesis, i.e. depression and mood disorders is caused by changes in the brain’s neurotrophic factors (Nestler et al., 2002 ▶).

Neurotrophins are the most important trophic factors in the nervous system that affect the proliferation, survival, and death of neural cells. The neurotrophin family includes nerve growth factor (NGF), brain-derived neurotrophic (BDNF), and neurotrophin-3 and neurotrophin-4 (NT3/4), (Binder and Scharfman, 2004 ▶). BDNF plays a regulatory role in neural differentiation (Jiao et al., 2014), synaptic plasticity (McAllister et al., 1999 ▶) and cell death through two cell surface receptors, which are TrkB and low-affinity nerve growth factor receptor (LNGFR) or P75 neurotrophin receptor (p75NTR) (Bamji et al., 1998 ▶). Studies have shown that direct injection of BDNF into the hippocampus and lateral ventricle of rodents exerts antidepressant effects in animal models (Shirayama et al., 2002 ▶). In addition, lower mRNA expression of *BDNF* and *TrkB* has been shown in the hippocampus of individuals with depression (Thompson Ray et al., 2011 ▶).

Cinnamomum has two species including *Cinnamomum zeylanicum Blume* and *Cinnamomum aromaticum Ness*. In the traditional medicine of Iran, China, and India, it is used for its exciting properties in treatment of respiratory disorders, gastrointestinal pain, and menstrual pain (Khan et al., 2003 ▶; Ouattara et al., 1997 ▶; Shen et al., 2010 ▶). In addition, previous studies have shown that HEC has an analgesic (Izadpanah et al., 2016 ▶), morphine withdrawal symptoms reducing (Moloudi et al., 2018 ▶) and anti-seizure effects (Ghaderkhani et al., 2014 ▶). Furthermore, the useful effects of HEC on reserpine-induced depression symptoms in mice were reported (Ghaderi et al., 2018 ▶). In this regards, anti-Alzheimer’s disease, anti-diabetic, skin-whitening, and antioxidant effects of the essential oil of *C. zeylanicum *were observed (Tepe and Ozaslan, 2020 ▶). Another study confirmed antioxidant activity of *C.zeylanicum *extracts (Mancini-Filho et al., 1998 ▶).

According to the published articles, the right prefrontal cortex is involved in negative emotions and the left prefrontal cortex is involved in positive emotions (Grimm et al., 2008 ▶; Murphy et al., 2003 ▶). Also, Ivamoto et al. chosed the right prefrontal cortex to evaluate the molecular mechanism of depression (Iwamoto et al., 2005 ▶). The majority of functional imaging studies showed prefrontal lobe hypometabolism in primary and secondary depression, with the severity of depression being often associated with the degree of frontal inactivity (George et al., 1994 ▶). 

It has also been shown that prefrontal dysfunction was associated with a poor antidepressant response in depressed elderly patients (Kalayam and Alexopoulos, 1999 ▶) and expression of depressive symptoms in patients with dementia of the Alzheimer's type (Akiyama et al., 2008 ▶).

Because of the above-mentioned effects of *Cinnamomum*, also due to the fact that cinnamon is widely used as an additive and since its underling mechanisms in depression have not been experimentally investigated, this study was designed to assess the effect of HEC on the BDNF protein and its receptor gene expression (*TrkB*) in prefrontal cortex of depressed rats.

## Materials and Methods

**Preparation of plant extract**

Cinnamon extraction was prepared from the bark of *C.zeylanicum*. The identification of the plant was done by a pharmacognosist from the School of Pharmacy, Hamedan University of Medical Sciences. Here, 100 g of cinnamon bark was ground into a thick paper filter, which was then loaded into the main chamber of the Soxhlet extractor. The solvent (ethanol 70%) was heated at a temperature above 50°C for 24 hr. To separate the extract and ethanol, it was placed in the rotary evaporator at a fixed temperature 40°C with a round 20 rpm for 1 hr.

**Animals and treatments**

Thirty-two male Wistar rats weighing 225–275 g were randomly selected and divided into 4 groups (n=8 rats per group) including: control group (received daily extract vehicle for four weeks in the absence of depression protocol), the depressed group (received daily extract vehicle, for four weeks and underwent depression protocol), Dep +HEC 200 group (received daily 200 mg/kg HEC, for four weeks and underwent depression protocol) and Dep +HEC 400 group (received daily 400 mg/kg HEC, for four weeks and underwent depression protocol). All injections were given intraperitoneally (ip). The vehicle was dimethyl sulfoxide (DMSO) 25% in normal saline.

**Depression protocol**

Animals in the control group did not receive any stimulation. The other three groups randomly received one of the following stimulations daily for 28 days: wet cage-floor for 24 hr, swimming in ice cold water (4°C) for 5 min, swimming in hot water (45°C) for 5 min, 45°cage tilting for 24 hr, day-night reversal for 24 hr, clamping the tail and shaking for 2 min, 30 times of electric shock at 1 mA for 10 sec, starvation for 48 hr, and deprivation from food and water for 24 hr. At the end of each week, behaviors of the animals were evaluated. The same stimulation was not be used consecutively (Huang et al. 2014 ▶).

**Sucrose preference test (SPT)**

After food and water deprivation for 24 hr, 1% sucrose solution was provided for rats before running the depression protocol as a base line. After starting the depression protocol, this test was repeated on days 7, 14, 21 and 28 to observe the amount of 1hr water or sucrose intake per 100 g body weight. SPT score was measured using the following equation, which evaluates the ratio of 1% sucrose solution consumed to the total liquid consumed: sucrose preference = sucrose consumed/ (sucrose consumed + tap water consumed) (Amiri et al., 2016 ▶).

**Forced swimming test (FST)**

Animals were placed in an open cylinder-shaped flask (diameter 40 cm, height 60 cm) filled with water (45 cm, 24±1°C). The behaviors of animals were recorded for 5 min. Swimming and immobility time were considered within the last 3 min. When animals stopped struggling and floated motionless on the water, it was regarded as “immobility” (Haj-Mirzaian et al., 2015 ▶).

**Tissue collection**

On the 29th day, animals were deeply anesthetized using a cocktail of 50 mg/kg ketamine and 4 mg/kg xylazine (ip), then they were sacrificed and the right prefrontal cortices were isolated on ice cold plates. Samples were immediately placed in liquid nitrogen and then frozen at -70°C. 

**Extraction of total RNA, cDNA synthesis and real time PCR**

RNA isolation was performed using the RNXTM-Plus reagent (Cinna Gen, Tehran) in accordance with the manufacturer’s instructions. RNA concentrations and quality were determined by absorbance readings at 260 nm using the photometric method. A reverse transcription was conducted by an easy cDNA synthesis kit (Cinna Gen, Tehran) using1 mM dNTPs mix, 3μg RNA adding 0.2μg random hexamer and appropriate amount of diethyl pyrocarbonate (DEPC)-treated water up to 10 μl. Afterwards, the mixture was incubated at 65ºC for 5 min and chilled on ice for 2 min. Finally, 10μl RT premix was added and reverse transcription was performed by incubation for 10 min at 25ºC followed by 60 min at 50ºC and the reaction was terminated by heating for 10 min at 70ºC. Real-Time PCR was done using the Corbett Rotor Gene 6000 Real Time PCR system (Corbett Research, Australia) and Syber Green Real Time PCR Master Mix (Pars Tous, Iran). The total volume was 20 μl, which contained 1μg cDNA, 1 pmol forward primer, 1 pmol reverse primer, 2X Syber Green PCR master mix (10μl), and dH_2_O (5μl). 

Real-time PCR was performed under following conditions: denaturation at 95°C for 15 min, 40 cycles of 15sec at 95°C, 57°C for 30sec, 72°C for 30 sec, and final extension at 72°C for10 min. The housekeeping gene beta-actin was used as an internal control. The gene expression ratio was obtained by P faffle method using PCR efficiency for each gene and ΔCT values. All PCR products were analyzed by a melting curve of the rotor-gene and on a 2% agarose gel. The primer sequences used were: 

Rat-*TrkB* Forward Primer: 5'-ATTTTGCACCAACCATCACAT-3 '(21bp)

Rat-*TrkB *Reverse Primer: 5'-AGCATCACCAGCAGGCAGA-3' (19bp)

β actin Forward (ACTBF)Primer: 5’-CACCCGCGAGTACAACCTTC-3’(20bp)

β actin Reverse (ACTBR)Primer: 5’-CCCATACCCACCATCACACC-3’(20bp)

**BDNF protein levels measured by enzyme-linked immunosorbent assay (ELISA)**

The tissue BDNF concentrations were assessed by the ELISA method using Millipore ELISA kit (Milipore, USA). Briefly, mouse BDNF-specific monoclonal antibodies were precoated onto the plates. BDNF and biotinylated mouse specific detection polyclonal antibodies were then added to the wells followed by the addition of Avidin-Biotin-Peroxidase Complex. Finally, horseradish peroxidase (HRP) substrate was used to visualize HRP enzymatic reaction and the concentration of BDNF was expressed as pg/ml. Based on the manufacturer's instructions; the limit of detection for this method was 15pg/ml.

**Statistical analysis**

The obtained data from the behavioral tests and gene expression ratios are given as the mean±SEM for 8 rats per group. The difference between the groups was determined by a one-way ANOVA followed by the Tukey's *post hoc *test. The trends of changes overtime were analyzed using Repeated measures ANOVA. The level of significance was set at p<0.05.

## Results

**The effect of HEC on SPT**

**Figure 1:**
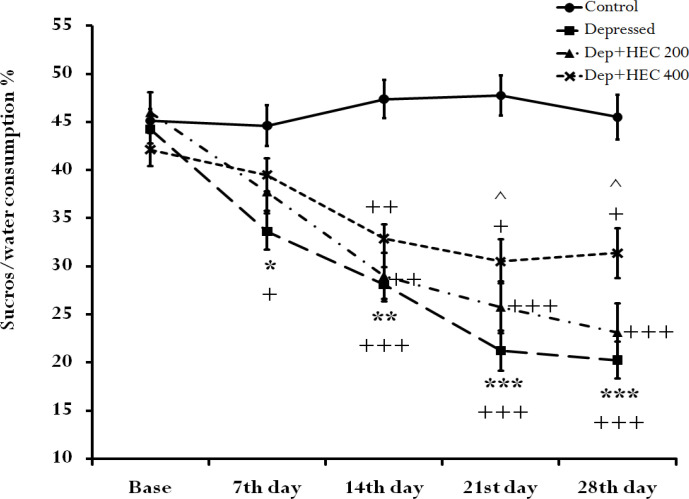
The SPT in control, depressed and Dep +HEC 200 and Dep +HEC 400 groups. Each marker is representing the mean±SEM for 8 rats. *p<0.05, **p<0.01 and ***p<0.001 show significant differences compared to the control group. +p<0.05, ++p<0.01 and^ +++^p<0.001 show significant differences within groups compared to the base. ^p<0.05 shows significant differences compared to the depressed group. Dep=depressed, HEC=hydroalcoholic extract of *Cinnamomum*, SPT=Sucrose preference test

The results shown in Figure 1 indicate a dramatic decrease in sucrose-water consumption in the depressed animals when compared to the control animals (P<0.05at 7^th^ day, p<0.01at 14^th^ day, p<0.001at 21^st^and 28^th^). In addition, the percentage of sucrose-water consumption on days 21 and 28 in the HEC 400 mg/kg group was significantly more than that of the depressed group (p<0.05). Also, in within-group comparison, the sucrose-water consumption trend was significantly decreased in depressed (p<0.05, at 7^th^ day and p<0.001, at 14^th^, 21^st^and 28^th^ days), Dep + HEC 200 (p<0.01 and p<0.001) and Dep + HEC 400 (p<0.05 and p<0.01) groups compared to their bases. But on days 21 and 28, in the Dep + HEC400 group, the downward trend decreased compared to the depressed group. 

**The effect of HEC on the swimming time index in the FST**

**Figure 2:**
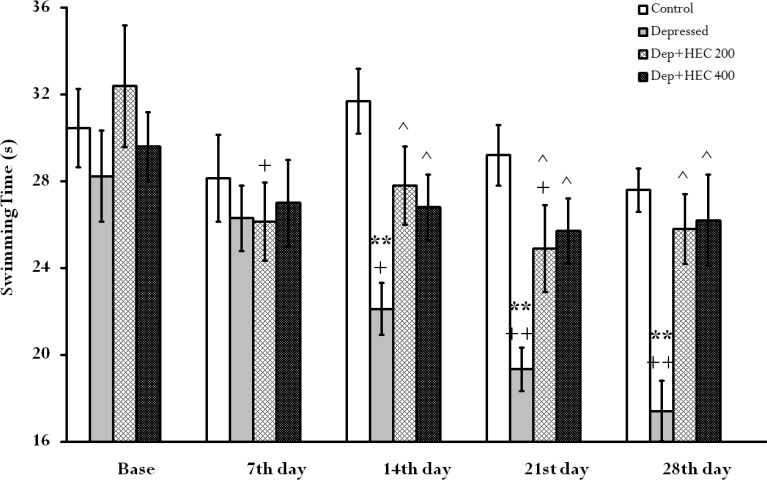
The swimming time index in the FST in the control, depressed and Dep +HEC 200 and Dep +HEC 400 groups. Each column is representing the mean±SEM for 8 rats. **p<0.01 shows significant differences compared to the control group. +p<0.05 and ++p<0.01 show significant differences within groups compared to the base. ^p<0.05 shows significant differences compared to the depressed group. Dep=depressed, HEC=hydroalcoholic extract of *Cinnamomum*

Figure 2 shows that the swimming time in the depressed group was significantly decreased on days 14, 21 and 28 compared to control group (p<0.01). On the other hand, HEC at the doses of 200 and 400 mg/kg prevented decreases in swimming time on days 14, 21 and 28compared to the depressed group (p<0.05). In addition, in within-group comparison, the swimming time trend was significantly decreased on days 14 (p< 0.05), 21 and 28 (p< 0.01) in depressed group compared to its base. While in the Dep + HEC 200 group, on days 7 and 21, there was a significant decrease compared to its base (p<0.05). 

**The effect of HEC on the immobility time in the FST**

**Figure 3:**
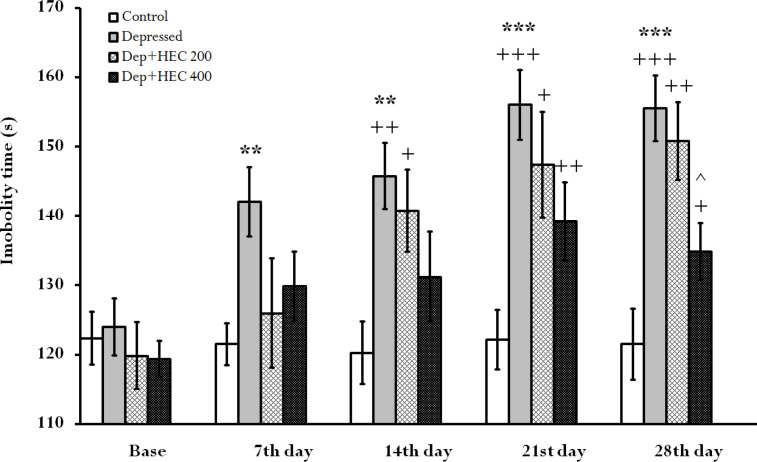
The immobility time index in the FST in the control, depressed and Dep +HEC 200 and Dep +HEC 400 groups. Each column is representing the mean±SEM for 8 rats. **p<0.01 and ***p<0.001 show significant differences compared to the control group. +p<0.05, ++p<0.01 and^ +++^p<0.001 show significant differences within groups compared to the base. ^p<0.05 shows significant differences compared with the depressed group. Dep=depressed, HEC=hydroalcoholic extract of *Cinnamomum*

The immobility time index in the depressed group on days 7, 14(p<0.01), 21 and 28 (p<0.001), was significantly increased compared to the control group. Furthermore, on the 28th day, this index was significantly reduced in the group treated with 400mg/kg HEC compared to the depressed group (p<0.05). In addition, in within-group comparison, the immobility time trend was significantly increased in the depressed (p<0.01 at 14^th^ day and at 21^st^ and 28^th^ days, p<0.001), Dep + HEC 200 (p<0.05 and p<0.01) and Dep + HEC 400 (p<0.05 and p<0.01) groups compared to their base. But on days 7 and 14, in the Dep +HEC 400 group, there was not a significant increase compared to the base (Figure 3).

**Effect of HEC on the BDNF protein level in the prefrontal cortex**

**Figure 4:**
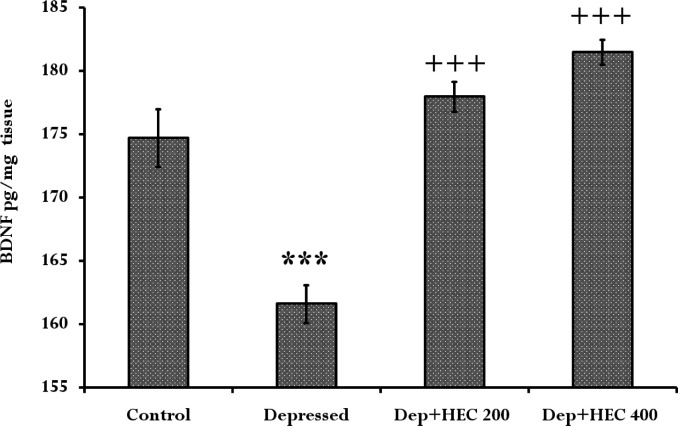
The BDNF protein level in the prefrontal cortex of the control, depressed and Dep +HEC 200 and Dep +HEC 400 groups. Each column represents the mean±SEM for 8 rats. ***p<0.001showsdifferences compared to the control group. ^+++^p<0.001 shows significant differences compared to the depressed group. Dep=depressed, HEC=hydroalcoholic extract of *Cinnamomum*

Figure 4 shows the results of the ELISA test indicating a significant decrease on BDNF protein levels in the depressed group when compared to the control group (p<0.001). But, treatment with HEC 200 and 400mg/kg significantly increased BDNF levels in comparison to the depressed group (p<0.001).

**Effect of HEC on** ***TrkB*** **gene expression in the prefrontal cortex**

**Figure 5:**
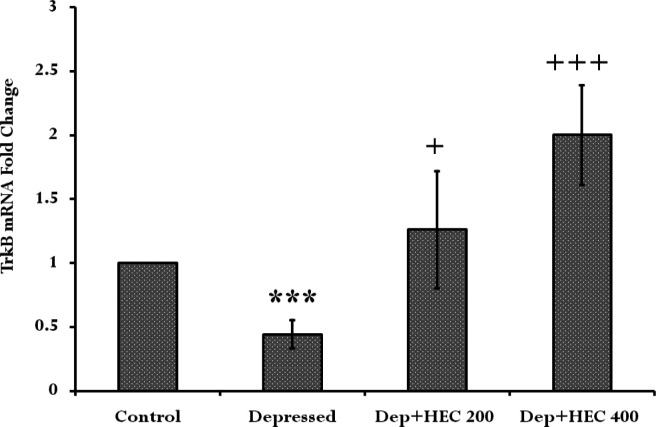
The *TrkB* gene expression ratio in the prefrontal cortex of the control, depressed and Dep +HEC 200 and Dep +HEC 400 groups. Each column represents the mean±SEM for 8 rats. ***p<0.001 shows significant differences compared to the control group. +p<0.05 and+++p<0.001 show significant differences compared to the depressed group. Dep=depressed, HEC=hydroalcoholic extract of *Cinnamomum*

The findings of real-time PCR showed that *TrkB* gene expression diminished significantly in the depressed group when compared to the control group (p<0.001). In addition, *TrkB* gene expression in the HEC-treated groups (200 and 400 mg/kg) was significantly higher than the depressed group (p<0.05, p<0.001; respectively) (Figure 5).

## Discussion

The results of the current study showed that induction of depression reduced swimming time, SPT, BDNF levels and *TrkB* gene expression, but increased immobility time compared to the control group. At the same time, administration of HEC significantly reversed these indices and trends. Trend analysis in Figures 1, 2 and 3 showed that long-term administration of the HEC has a greater antidepressant effect, which is probably due to the mechanism of action of the extract through modulation of *TrkB* gene or BDNF expression. 

In support, Rabadia et al. assessed the antidepressant effect of *Cinnamomum camphora* oil in mice by using forced swimming and tail suspension tests. Their results showed that camphor oil significantly decreased immobility time. They attributed this antidepressant effect to terpene and monoterpenoid compounds such as beta-pinene, beta-thujone, limonene, and linalool (Rabadia et al., 2013 ▶). These compounds are the main ingredients of *C. zeylanicum *(Ranasinghe et al., 2013 ▶; Rao and Gan, 2014 ▶) which have anti-depressant properties mediated by inhibiting monoamine oxidase A and B (Van Diermen et al., 2009 ▶). Also, *C. zeylanicum*, with its beta-pinene, can increase the activity of the animal by increasing the level of dopamine and reducing the activity of monoamine oxidase (Li et al., 2018 ▶). 

One of the most obvious neuroanatomical changes in depression is a decrease in the size of the hippocampus in depressed individuals, and it has been shown that some antidepressants play an important role in cell regeneration by stimulating neurotrophic factors expression. The most important growth factor that affects the growth of brain cells is BDNF which is increased by antidepressants in the hippocampus (Ashwani and Preeti, 2012 ▶; Hackley, 2010 ▶). Cinnamon contains compounds such as cinnamaldehyde and eugenol. Eugenol has a special effect on improving symptoms of the central nervous system diseases such as Alzheimer's, Parkinson's, and depression. Previous studies have shown that eugenol increased the expression of neurotrophic factors in the hippocampus that led to brain cell regeneration. In addition, eugenol can increase metallothionein-3, a neuroprotective protein, gene expression in the hippocampus. Therefore, the antidepressant effects of cinnamon may be related to eugenol. Furthermore, cinnamaldehyde plays an important role in pain relief and stimulation of the central nervous system (Alqasoumi, 2012 ▶).

Studies suggest that natural commands can be useful for treatment of Parkinson's, Alzheimer's, and depression. Therefore, this study has confirmed the antidepressant effects of cinnamon (Null et al., 2017 ▶). Masi and Brovedani demonstrated that neurotrophic factors such as BDNF play a major role in the development of depression. Neurotrophic factor levels in depressed patients reduced and decreased neurogenesis in the hippocampus exacerbated the symptoms of depression. Additionally, anti-depressant agents can increase serum BDNF levels via CREB protein phosphorylation (Masi and Brovedani, 2011 ▶). The results of Rabie et al. study showed that patients with bipolar disorders had low levels of BDNF in their brain; however, after treatment, BDNF levels gradually increased and decreased disease symptoms. The results of this study showed that BDNF can be used as a marker of disease progression in patients with mood disorders (Rabie et al., 2014 ▶). In support of neurotrophic factor theory, our results showed a significant decrease of BDNF levels in the depressed group when compared to the control group. In addition, BDNF levels in depressed HEC–treated groups were significantly increased compared to the depressed group. These results confirmed mood improvement by increased BDNF level. In line with our study, Jana et al reported that two species of cinnamon (*C.cassia* and *C.verum*) can increase the levels of BDNF in the cerebral cortex (Jana et al., 2013 ▶). Also, sodium benzoate (NaB) the metabolite of cinnamon, could increase the levels of BDNF in human astrocytic cell cultures. It has been proven that the glutamate release in the CNS improves mood and activates receptors that can increase BDNF and NGF levels in the hippocampus (Govitrapong et al., 1998 ▶). Also, treatment of chronic depression can increase the BDNF mRNA expression in the hippocampus. Also, injecting BDNF into the hippocampus and midbrain has effects similar to antidepressant agents (Saarelainen et al., 2003 ▶). Taken together, these studies confirm the possible effect of cinnamon on mood elevation through increases in BDNF and its receptor gene expressions. On the other hand, our results showed a significant increase in *TrkB* receptor gene expression in the depressed groups receiving HEC when compared to the depressed group. McAllister et al showed that BDNF receptors via a variety of signaling cascades, can lead to neurogenesis, synaptic plasticity, and suppression of apoptosis. BDNF increases neuronal proliferation, axonal conductance, dendrites growth, and release of neurotransmitters. In addition, they showed that in severe depression, the size of hippocampus and the prefrontal cortex is significantly reduced which can be the cause of depressive illnesses (McAllister et al., 1996 ▶). In line with our results Zhou et al. showed that, antidepressant-like effects of biperiden were antagonized by pretreatment with the *TrkB* antagonist K252a (Zhou et al., 2017 ▶). Furthermore, Ma et al showed that *TrkB* antagonist ANA-12 significantly blocked beneficial combination effects of brexpiprazole and fluoxetine on rapid antidepressant action via BDNF-TrkB signaling pathway (Ma et al., 2016 ▶).

The results of the present study showed that HEC reduced the symptoms of depression in rats. These effectswere probably exerted through induction of the BDNF protein and its receptor, *TrkB* gene expression.

## References

[B1] Akiyama H, Hashimoto H, Kawabe J, Higashiyama S, Kai T, Kataoka K, Shimada A, Inoue K, Shiomi S, Kiriike N (2008). The relationship between depressive symptoms and prefrontal hypoperfusion demonstrated by eZIS in patients with DAT. Neurosci lett.

[B2] Alqasoumi S (2012). Anti-secretagogue and antiulcer effects of Cinnamon Cinnamomum zeylanicum in rats. J. Pharmacogn Phytother.

[B3] Amiri S, Amini-Khoei H, Mohammadi-Asl A, Alijanpour S, Haj-Mirzaian A, Rahimi-Balaei M, Razmi A, Olson, CO, Rastegar M, Mehdizadeh, M, Zarrindast MR (2016). Involvement of D1 and D2 dopamine receptors in the antidepressant-like effects of selegiline in maternal separation model of mouse. Physiol Behav.

[B4] Andrade L, Caraveo-Anduaga JJ, Berglund P, Bijl RV, De Graaf R, Vollebergh W, Dragomirecka E, Kohn R, Keller M, Kessler RC, Kawakami N, Kilic C, Offord D, Ustun TB, Wittchen HU (2003). The epidemiology of major depressive episodes: results from the International Consortium of Psychiatric Epidemiology (ICPE) Surveys. Int J Methods Psychiatr Res.

[B5] Ashwani A, Preeti V (2012). A review of pathophysiology, classification and long term course of depression. IRJP.

[B6] Bamji SX, Majdan M, Pozniak CD, Belliveau DJ, Aloyz R, Kohn J, Causing CG, Miller FD (1998). The p75 neurotrophin receptor mediates neuronal apoptosis and is essential for naturally occurring sympathetic neuron death. J Cell Biol.

[B7] Binder DK, Scharfman HE (2004). Brain-derived neurotrophic factor. Growth Factors.

[B8] Carney RM, Freedland KE (2017). Depression and coronary heart disease. Nat Rev Cardiol.

[B9] George MS, Ketter TA, Post RM (1994). Prefrontal cortex dysfunction in clinical depression. Depression.

[B10] Ghaderi H, Rafieian M, Nezhad H (2018). Effect of hydroalcoholic Cinnamomum zeylanicum extract on reserpine-induced depression symptoms in mice. Pharmacophore.

[B11] Ghaderkhani S, Moloudi MR, Izadpanah E, Mohammadi R, Rostami A, Khomand P, Hassanzadeh K (2014). Effect of hydroalcoholic extract of cinnamomum on strychnine-induced seizure in mice. J Isfahan Med Sch.

[B12] Govitrapong P, Suttitum T, Kotchabhakdi N, Uneklabh T (1998). Alterations of immune functions in heroin addicts and heroin withdrawal subjects. J Pharmacol Exp Ther.

[B13] Grimm S, Beck J, Schuepbach D, Hell D, Boesiger P, Bermpohl F, Niehaus L, Boeker H, Northoff G (2008). Imbalance between left and right dorsolateral prefrontal cortex in major depression is linked to negative emotional judgment: an fMRI study in severe major depressive disorder. Biol Psychiatry.

[B14] Hackley B (2010). Antidepressant medication use in pregnancy. J Midwifery Womens Health.

[B15] Haj-Mirzaian A, Amiri S, Kordjazy N, Rahimi-Balaei M, Haj-Mirzaian, A, Marzban H, Aminzadeh A, Dehpour AR, Mehr SE (2015). Blockade of NMDA receptors reverses the depressant, but not anxiogenic effect of adolescence social isolation in mice. Eur J Pharmacol.

[B16] Huang X, Mao YS, Li C, Wang H, Ji JL (2014). Venlafaxine inhibits apoptosis of hippocampal neurons by up-regulating brain-derived neurotrophic factor in a rat depression model. Int J Clin Exp Pathol.

[B17] Iwamoto K, Nakatani N, Bundo M, Yoshikawa T, Kato T (2005). Altered RNA editing of serotonin 2C receptor in a rat model of depression. Neurosci Res.

[B18] Izadpanah E, Nikandam F, Moloudi MR, Hassanzadeh K (2016). Evaluation of the analgesic effect of hydroalcoholic extract of Cinnamomum in rats. SJKUMS.

[B19] Jana A, Modi K, Roy A, Anderso J, van Breemen R, Pahan K (2013). Up-Regulation of Neurotrophic Factors by Cinnamon and its Metabolite Sodium Benzoate: Therapeutic Implications for Neurodegenerative Disorders. JNIP.

[B20] Jiao Y, Palmgren B, Novozhilova E, Englund Johansson U, Spieles-Engemann AL, Kale A, Stupp SI, Olivius P (2014). BDNF increases survival and neuronal differentiation of human neural precursor cells cotransplanted with a nanofiber gel to the auditory nerve in a rat model of neuronal damage. Biomed Res Int.

[B21] Kalayam B, Alexopoulos GS (1999). Prefrontal dysfunction and treatment response in geriatric depression. Arch Gen Psychiatry.

[B22] Khan A, Safdar M, Ali Khan MM, Khattak KN, Anderson RA (2003). Cinnamon improves glucose and lipids of people with type 2 diabetes. Diabetes Care.

[B23] Li YR, Fu CS, Yang WJ, Wang XL, Feng D, Wang XN, Ren DM, Lou HX, Shen T (2018). Investigation of constituents from Cinnamomum camphora ( J Presl and evaluation of their anti-inflammatory properties in lipopolysaccharide-stimulated RAW 264 7 macrophages. J Ethnopharmacol.

[B24] Ma M, Ren Q, Yang C, Zhang Jc, Yao W, Dong C, Ohgi Y, Futamura T, Hashimoto K (2016). Adjunctive treatment of brexpiprazole with fluoxetine shows a rapid antidepressant effect in social defeat stress model: Role of BDNF-TrkB signaling. Sci Rep.

[B25] Mancini-Filho J, Van-Koiij A, Mancini D, Cozzolino F, Torres R (1998). Antioxidant activity of cinnamon (Cinnamomum Zeylanicum, Breyne) extracts. Bollettino chimico farmaceutico.

[B26] Mansson D (2019). Looking to the future of depression: instant diagnosis and medication-free treatment. Future science OA.

[B27] Masi G, Brovedani P (2011). The hippocampus, neurotrophic factors and depression: possible implications for the pharmacotherapy of depression. CNS Drugs.

[B28] McAllister AK, Katz LC, Lo DC (1996). Neurotrophin regulation of cortical dendritic growth requires activity. Neuron.

[B29] McAllister AK, Katz LC, Lo DC (1999). Neurotrophins and synaptic plasticity. Annu Rev Neurosci.

[B30] Moinuddin G, Devi K, Khajuria DK (2012). Evaluation of the anti–depressant activity of Myristica fragrans (Nutmeg) in male rats. Avicenna J. Phytomedicine.

[B31] Moloudi MR, Moqbel H, Dastan D, Hasanzadeh K, Noori B, Izadpanah E (2018). Effect of Hydro-alcoholic extract of Jasminum sambac on morphine withdrawal symptoms in rats. SJKUMS.

[B32] Montazeri A, Mousavi SJ, Omidvari S, Tavousi M, Hashemi A, Rostami T (2013). Depression in Iran: a systematic review of the literature (2000_2010). Payesh.

[B33] Murphy FC, Nimmo-Smith I, Lawrence, AD (2003). Functional neuroanatomy of emotions: a meta-analysis. Cogn Affect Behav Neurosci.

[B34] Nestler E, Barrot M, DiLeone R, Eisch A, Gold S, Monteggia L (2002). Neurobiology of Depression. Neuron.

[B35] Null G, Pennesi L, Feldman M (2017). Nutrition and Lifestyle Intervention on Mood and Neurological Disorders. Evid Based Complementary Altern Med.

[B36] Ouattara B, Simard RE, Holley RA, Piette GJ, Begin A (1997). Antibacterial activity of selected fatty acids and essential oils against six meat spoilage organisms. Int J Food Microbiol.

[B37] Patro G, Bhattamisra SK, Mohanty BK (2016). Effects of Mimosa pudica L leaves extract on anxiety, depression and memory. Avicenna J. Phytomedicine.

[B38] Rabadia J, Satish S, Ramanjaneyulu V (2013). "An Investigation of Anti-Depressant Activity of Cinnamomum Camphora Oil in Experimental Mice ". Asian j biomed pharm.

[B39] Rabie MA, Mohsen M, Ibrahim M, El-Sawy Mahmoud R (2014). Serum level of brain derived neurotrophic factor (BDNF) among patients with bipolar disorder. J Affect Disord.

[B40] Ranasinghe P, Pigera S, Premakumara GA, Galappaththy P, Constantine GR, Katulanda P (2013). Medicinal properties of 'true' cinnamon (Cinnamomum zeylanicum): a systematic review. BMC Complement Altern Med.

[B41] Rao PV, Gan SH (2014). Cinnamon: a multifaceted medicinal plant. Evid Based Complement Alternat Med.

[B42] Saarelainen T, Hendolin P, Lucas G, Koponen E, Sairanen M, MacDonald E, Agerman K, Haapasalo A, Nawa H, Aloyz R, Ernfors P, Castren E (2003). Activation of the TrkB neurotrophin receptor is induced by antidepressant drugs and is required for antidepressant-induced behavioral effects. J Neurosci.

[B43] Shen Y, Fukushima M, Ito Y, Muraki E, Hosono T, Seki T, Ariga T (2010). Verification of the antidiabetic effects of cinnamon (Cinnamomum zeylanicum) using insulin-uncontrolled type 1 diabetic rats and cultured adipocytes. Biosci Biotechnol Biochem.

[B44] Shirayama Y, Chen AC, Nakagawa S, Russell DS, Duman RS (2002). Brain-derived neurotrophic factor produces antidepressant effects in behavioral models of depression. J Neurosci.

[B45] Steptoe A, Tsuda A, Tanaka Y, Wardle J (2007). Depressive symptoms, socio-economic background, sense of control, and cultural factors in university students from 23 countries. Int J Behav Med.

[B46] Tepe AS, Ozaslan M (2020). Anti-alzheimer, anti-diabetic, skin-whitening, and antioxidant activities of the essential oil of Cinnamomum zeylanicum. Ind Crops Prod.

[B47] Thompson Ray M, Weickert CS, Wyatt E, Webster MJ (2011). Decreased BDNF, trkB-TK+ and GAD67 mRNA expression in the hippocampus of individuals with schizophrenia and mood disorders. J Psychiatry Neurosci.

[B48] Van Diermen D, Marston A, Bravo J, Reist M, Carrupt PA, Hostettmann K (2009). Monoamine oxidase inhibition by Rhodiola rosea L. roots. J Ethnopharmacol.

[B49] Zhou D, Zhang Z, Liu L, Li C, Li M, Yu H, Cai X, Sun X, Shen X, Wang J, Geng J, Wang C, Shi Y (2017). The antidepressant-like effects of biperiden may involve BDNF/TrkB signaling-mediated BICC1 expression in the hippocampus and prefrontal cortex of mice. Pharmacol Biochem Behav.

